# Gastric cancer cell types display distinct proteasome/immunoproteasome patterns associated with migration and resistance to proteasome inhibitors

**DOI:** 10.1007/s00432-023-04948-z

**Published:** 2023-06-01

**Authors:** Francesca Monittola, Marzia Bianchi, Maria Gemma Nasoni, Francesca Luchetti, Mauro Magnani, Rita Crinelli

**Affiliations:** Department of Biomolecular Sciences, University of Urbino Carlo Bo, 61029 Urbino, PU Italy

**Keywords:** Proteasome, Immunoproteasome, Gastric cancer, Cell migration, Proteasome inhibitors, 20S regulatory particles

## Abstract

**Purpose:**

Gastric cancers (GC) display histological and molecular differences. This heterogeneity has limited the development of new therapeutic strategies which requires the identification of the molecular players involved in GC pathogenesis and the investigation of their responsiveness to drugs. Several proteasome subunits have been identified as prognostic markers in GC and their role studied by gene knockdown. However, proteasomes are multi-subunit protein complexes co-existing in multiple forms with distinct activity/specificity and ability to change in response to inhibitors. Information on the role of different proteasome particles in cancer and their relevance as therapeutic targets is limited.

**Methods:**

Based on this evidence, subunit assembly into proteasome complexes and activity were investigated by native PAGE followed by immunoblotting, and by using fluorogenic substrates, respectively.

**Results:**

Here we show that GC cell lines with epithelial and/or diffuse Lauren’s histotype express different levels of immunoproteasome subunits and equal amounts of constitutive counterparts. Immunoproteasome subunits were highly expressed and preferentially assembled into 19S capped complexes in diffuse-type cells, where most of the activity was catalyzed by the 26S and 30S particles. In epithelial cells, activity appeared equally distributed between 19S- and 11S-capped proteolytic particles. This proteasome pattern was associated with higher resistance of diffuse-type cells to proteasome inhibition. Immunoproteasome inhibition by ONX 0914 did not influence cell viability but affected metastatic cell migration.

**Conclusions:**

These results suggest that pharmacological inhibition of the immunoproteasome may be useful in treating metastatic gastric cancers.

**Supplementary Information:**

The online version contains supplementary material available at 10.1007/s00432-023-04948-z.

## Introduction

The multicatalytic proteasome complex plays an essential role in tumor initiation and progression (Jang 2018). For this reason, it is considered a drug target and a potential diagnostic and prognostic biomarker in cancer (Fricker 2020). The proteasome is a large multi-subunit complex; its proteolytic activity relies on the following three subunits: β1 (PSMB6), β2 (PSMB7) and β5 (PSMB5), which are incorporated into the so-called constitutive proteasome (CP). Subunits β1i (PSMB9 or LMP2), β2i (PSMB10) and β5i (PSMB8 or LMP7) replace the former in the immunoproteasome (IP), a specialized variant of the proteasome (Tanaka 2013; Bard et al. 2018). Catalytic and non-catalytic β subunits (β 1–7) are assembled to form two identical β rings which are stacked and capped on both sides by α subunit rings, building up the αββα core particle 20S. Three following activities are associated with CP: caspase-like (or peptidyl-glutamyl peptide-hydrolyzing-like), trypsin-like and chymotrypsin-like. Compared to CP the IP possesses enhanced chymotrypsin- and trypsin-like activities and reduced caspase-like activity, making it more suitable for processing major histocompatibility complex I peptides (Gaczynska et al. 1993). Intermediate or mixed-type proteasome variants, containing an assortment of β and βi subunits, have been described in normal and cancer tissues and cells (Dahlmann 2016; Morozov and Karpov 2019). The most common configurations are β1/β2/β5i and β1i/β2/β5i with increased chymotrypsin- and trypsin-like activity and, in the second case, also reduced caspase-like activity (Guillaume et al. 2010). The activity of the 20S complex is further regulated by two main regulatory complexes which can transiently associate with the core particle influencing substrate degradation rates and selectivity as follows: the 19S regulator, forming the 30S (double-capped) and 26S (single-capped) proteasomes, and the 11S or PA28. Both regulators can associate with the constitutive and immunoproteasome 20S core (i20S) (Fabre et al. 2015).

The immunoproteasome is constitutively expressed in immune cells, where it is implicated in cell activation and inflammatory cytokine production while it is induced in non-immune cells (Groettrup et al. 2010). Interestingly, high levels of immunoproteasome expression have been found in some tumor types raising the question of whether the immunoproteasome may serve other functions. In tumor cells immunoproteasome expression has been attributed to paracrine mechanisms and found correlated to pro-tumorigenic cytokine and chemokine production but also to increased presentation of tumor peptides with improved immune surveillance (Tripathi et al 2021). However, constitutive immunoproteasome expression without stimulation has been detected in several cancer cells, indicating that these cells may rely on immunoproteasome function for their basal metabolism/survival (Rouette et al. 2016). The functional relevance of the immunoproteasome in cancerous cells is poorly understood (Tripathi et al 2021).

We have previously demonstrated that gastric cancer (GC) cell lines display different ubiquitin (Ub) gene expression pattern and sensitivity to ubiquitin knockdown (Scarpa et al. 2020). GC cell lines are similar for Ub content but differ for the chymotrypsin-like proteasome activity. This evidence prompted us to dissect the molecular bases of such difference providing further insight into the Ub/proteasome system in GC.

Kwon et al (2016) identified the PSMB8 gene encoding β5i subunit as a biomarker of gastric cancer poor prognosis and as a critical player in GC cell migration and invasion. In this paper we provide evidence that GC cells express all the three immunoproteasome subunits together with their constitutive counterparts. Expression of catalytic βi subunits was higher in diffuse-type KATO III cells, with preferential incorporation into 19S capped complexes, compared to intestinal-type 23132/87 cells. MKN45 cells with a mixed intestinal/diffuse phenotype displayed proteasome features of both histotypes.19S capping of the proteasome was associated with higher resistance to inhibition of cell proliferation by proteasome/immunoproteasome inhibitors. By contrast, immunoproteasome inhibition by ONX 0914 strongly affected cell migration of metastatic cell lines.

Overall, these findings indicate that GC cell proteasomal repertoire is more heterogeneous than previously assumed based on gene expression analyses.

## Materials and methods

### Cell culture and treatment

23132/87 and MKN45 were purchased from DMSZ (German Collection of 418 Microorganisms and Cell Cultures), while KATO III were from ATCC (American Type Culture Collection). Cells were periodically tested for mycoplasma contamination by PCR analysis and authenticated by Short Tandem Repeat (STR) DNA Genotype analysis and Cellosaurus database comparison. Cells were grown in FBS/antibiotic supplemented RPMI 1640 medium (SIGMA Aldrich) (Scarpa et al. 2020). Bortezomib and ONX 0914 (Cayman Chemicals) were dissolved in dimethyl sulfoxide (DMSO). Cycloheximide (CHX) was suspended in ethanol. The final concentration of the vehicle in the culture medium never exceeded 0.02% (v/v).

### Cell lysates, SDS PAGE and western immunoblotting analysis

Cells were directly lysed on petri dishes with a denaturing buffer consisting of 50 mM Tris–HCl, pH 7.8, 0.25 M sucrose, 2% (w/v) sodium dodecyl sulphate (SDS), 10 mM N-ethylmaleimide, supplemented with a cocktail of protease (Complete, Roche) and phosphatase inhibitors (1 mM NaF, 1 mM Na_3_VO_4_). Samples were heated at 100 °C, sonicated at 70 Watts for 40 s and centrifuged at 14,000 × *g* to remove debris. Proteins were separated by SDS PAGE (polyacrylamide gel electrophoresis) and immunoblotted onto PVDF (polyvinylidene difluoride) 0.2 µm pore size membrane. After transfer, proteins were visualized on the membrane with the No-Stain^™^ Protein Labeling Reagent (Invitrogen) and then stained with the following primary antibodies: proteasome 20S α1, 2, 3, 5, 6 and 7 subunits (pan α) (MCP231) (#PW8195, Enzo Life Sciences), PSMB4/β7 (#A303-819A-T, Bethyl Laboratories), 20S proteasome β1 (D-9) (#sc-374405, Santa Cruz Biotechnology), PSMB5/β5 (#ALS17241, Abcepta), PSMB9/β1i (#AP21207b, Abcepta), PSMB8/β5i (#13635, Cell Signaling Technology), PSMB7/β2 (#14771, ABclonal), PSMB10/β2i (#A5452, ABclonal), PSMC6/ATPase6 (#A5377, ABclonal), ubiquitin (kindly provided by Prof. A.L. Haas, New Orleans School of Medicine). In some experiments β-actin rabbit polyclonal antibody (#4967, Cell Signaling Technology) was used to check equal protein loading. Immunoreactive bands were detected by horseradish peroxidase-conjugate secondary antibody (BioRad laboratories Inc.) and the enhanced chemiluminescence detection kit WesternBright ECL (Advasta) in a ChemiDoc MP Imaging System (Bio-Rad). Quantification of the immunoreactive bands was performed using the Image Lab analysis software version 5.2.1 (Bio-Rad).

### RNA preparation and quantitative real-time PCR (qPCR)

Total RNA was extracted using the RNeasy Plus Mini kit (Qiagen Inc.). RNA (500 ng) was reverse transcribed using PrimeScript™ RT Master Mix (Perfect Real Time; Takara Bio Europe SAS). cDNA was amplified using the Hot-Rescue Real Time PCR Kit (Diatheva s.r.l.) and visualized with SYBR Green using an ABI PRISM 7500 Sequence detection system (Applied Biosystems). Thermal cycling was performed as follows: 10 min at 95 °C; 40 cycles of denaturation at 95 °C for 15 s, annealing at 60 °C for 15 s, and extension at 72 °C for 30 s. Expression data were calculated according to the 2^−ΔΔCt^ method (Livak and Schmittgen 2001). The primers used are the following: β2M (beta 2-microglobulin) Fwd: GCCTGCCGTGTGAACCAT, Rev: CATCTTCAAACCTCCATGATGCT; PSMB8 (β5i) Fwd: GACAGTGGCTATCGGCCTAA, Rev: TCACCCAACCATCTTCCTTC; PSMB9 (β1i) Fwd: CAACGTGAAGGAGGTCAGGT, Rev: TGCTGCATCCACATAACCAT; PSMB10 (β2i) Fwd: ATACGCGAGCCACTAACGAT, Rev: CAGCCCCACAGCAGTAGATT; PSMB5 (β5) Fwd: ACGTGGACAGTGAAGGGAAC, Rev: CTGCTCCACTTCCAGGTCAT; PSMB6 (β1) Fwd: CAGAACAACCACTGGGTCCT, Rev: TGGTAGGTGACAGCATCAGC; PSMB7 (β2) Fwd: GCAACTGAAGGGATGGTTGT, Rev: GCTGGGTTGTCATGTCTGTG; PSMB4 (β7) Fwd: GAGCTTCCTCGGTTATGTGG, Rev: GCTTAGCACTGGCTGCTTCT.

### Native PAGE analysis of proteasome complexes

Native gel analysis of proteasome complexes was performed as described in Yazgili et al (2021) and Roelofs et al (2018). GC cells were lysed in native buffer consisting of 10 mM Tris–HCl pH 7.5, 5 mM MgCl_2_, 10 mM NaCl, 10% (v/v) glycerol, 1 mM dithiotreithol (DTT), 2 mM ATP, and a cocktail of protease inhibitors, by seven freezing and thawing cycles in liquid nitrogen. Lysates were then centrifuged at 20,000 × *g* at 4 °C. 15–20 µg of proteins were loaded on 4% (w/v) polyacrylamide gels in Tris/Borate buffer containing 0.5 mM ATP. Gels were run at 150 V for 2.5 h at + 4 °C. In gel proteasome activity was performed as described in Yazgili et al (2021) by incubating the gel in reaction buffer (50 mM Tris–HCl pH 7.5, 1 mM ATP, 10 mM MgCl_2_, 1 mM DTT, 50 μM fluorogenic substrate) for 30 min at 37 °C in the dark. Images were visualized in a Gel Doc system (Bio Rad) under UV light. After denaturation in solubilization buffer (2% SDS, 66 mM Na_2_CO_3_, 1.5% β-mercaptoethanol) for 20 min at room temperature, proteins were blotted onto PVDF membranes and immunostained with antibodies against proteasome subunits.

### Proteasome activity assay

Proteasome activity was measured in native cell extracts obtained as described above using the following synthetic fluorogenic substrates (Cayman Chemicals): s-LLVY-AMC (Suc-Leu-Leu-Val-Tyr-7-amido-4-methylcoumarin) for the chymotrypsin-like activity; Boc-LRR-AMC (Boc-Leu-Arg-Arg- AMC) for the trypsin-like activity; Z-LLE-AMC (Z-Leu-Leu-Glu-AMC) for the caspase-like activity. Ac-ANW-AMC (Ac-Ala-Asn-Trp-AMC) and Ac-PAL-AMC (Ac-Pro-Ala-Leu-AMC) were used as β5i and β1i specific substrate, respectively. The assay buffer consisted of 50 mM Hepes/KOH pH 7.8, 10 mM KCl, 2.5 mM ATP and 25 mM MgCl_2_. The reaction was initiated by addition of the fluorogenic peptide (200 μM s-LLVY-AMC, Z-LLE-AMC and Boc-LRR-AMC; 100 μM Ac-ANW-AMC and 20 μM Ac-PAL-AMC). Protein extract concentration was within the range of the linear signal-concentration response, typically between 0.2 and 0.1 mg/ml. The release of AMC from peptidyl derivatives after hydrolysis was measured at 37 °C for 30 min with an excitation/emission wavelengths of 355/460 nm. Proteasome activity was calculated from the slope after linear regression analysis of the values plotted as a function of time (*R*^2^ > 0.95).

### Cell viability assay

Cells (25,000 cells/well) were seeded in 96-well plates. After 24 h, cells were treated with proteasome inhibitors or with the vehicle DMSO. Each concentration was tested in triplicates. Cell viability was evaluated 24–48 h post-treatment by using the EZMTT^™^ Cell Proliferation Assay reagent (Merck Millipore).

### Transwell migration assay

Cells were seeded into the trans-well upper chamber (12-well cell, 8.0 µm insert, CellQUART, SabeuGmbH & Co, Germany) at a density of 1.5 × 10^5^ cells/well in 500 µL serum-free medium. The lower chamber was filled with 1 ml complete medium containing ONX 0914 or the vehicle as control. After 24 h, migrating cells were stained with 1 µM calcein-AM (Molecular Probes, Eugene, OR, USA) for 30 min at 37 °C. The images were obtained using a digital camera-attached fluorescence microscope with data acquisition software (Nikon ECLIPSE TS100, software NIS-Elements F, Nikon). The number of migrating cells was quantified in 10 fields randomly selected.

### Statistical analysis

Data were analyzed using Prism software version 5.0 (GraphPad). One-way ANOVA was used to compare the experimental groups followed by the Tukey’s test (multiple comparisons) or the Dunnet’s test (versus the control group mean).

## Results

### GC cells co-express constitutive and immunoproteasome subunits

We have previously demonstrated that MKN45 contained a significantly higher 20S proteasome activity compared to 23132/87 cells as determined by assessing the chymotrypsin-like activity with the commonly used fluorogenic substrate s-LLVY-AMC (Scarpa et al. 2020). Based on this evidence, we decided to characterize the proteasome pattern in GC cells. A third GC cell line, KATO III, was included in this study. The three cell lines are representative of different histotypes: 23132/87 intestinal; MKN45 intestinal/diffuse, and KATO III diffuse type based on the Lauren’s classification (Lauren 1965; Motoyama et al. 1986). Moreover, 23132/87 cells are derived from a primary tumor, while MKN45 and KATO III are metastatic, thus undifferentiated.

**Fig. 1:**
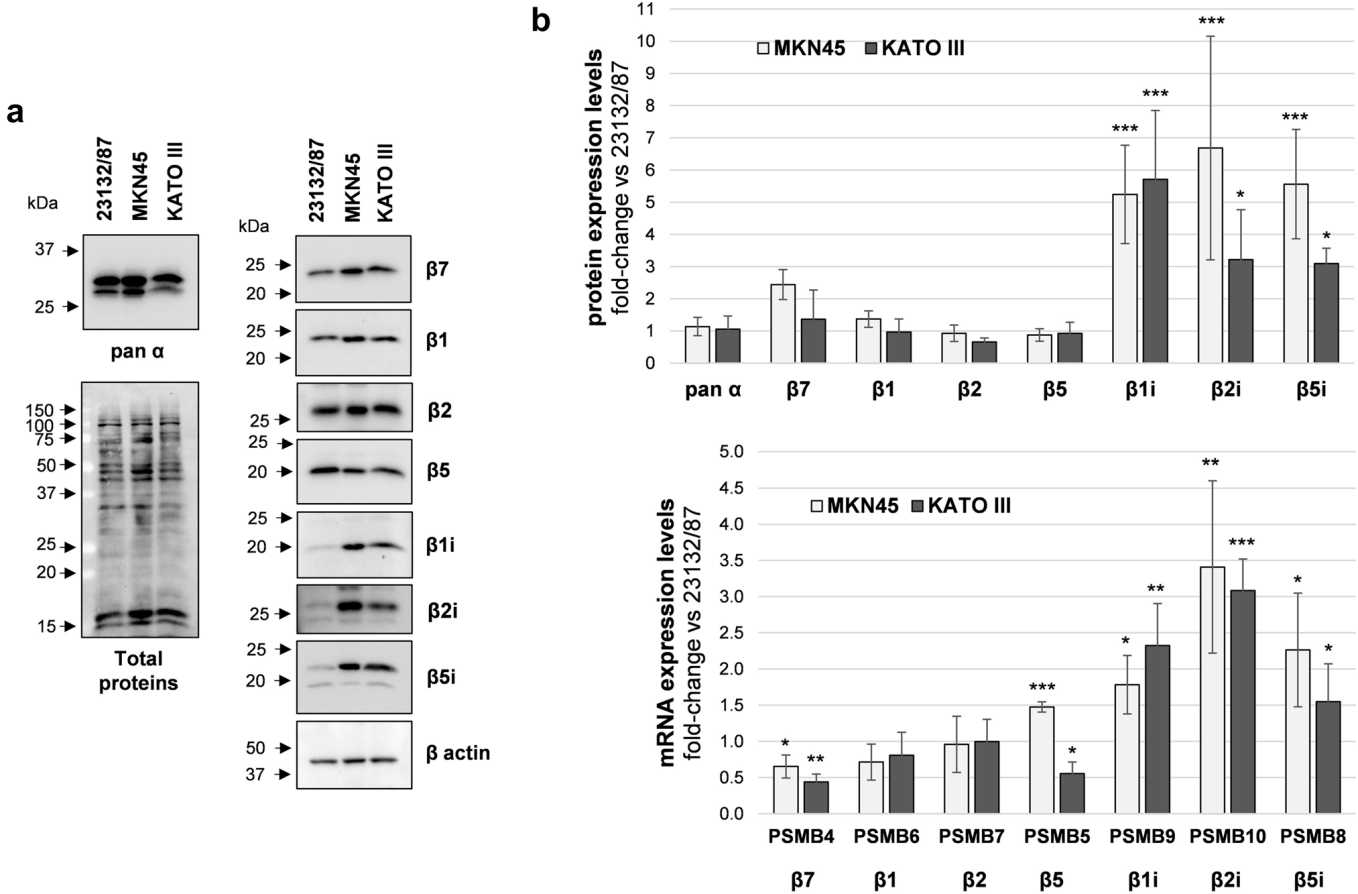
Proteasome and immunoproteasome subunit expression in GC cell lines. **a** Representative images of proteasome subunit levels in GC cells; total proteins and β-actin were stained as loading controls. Whole cell extracts were separated onto 12% (w/v) polyacrylamide gels (2.5 µg/lane). After SDS-PAGE, proteins were immunoblotted and stained with the indicated antibodies. Total proteins were visualized on the membrane with the No stain labeling reagent. Image acquisition was performed in a ChemiDoc system. On the left, arrows indicate the position of molecular weight markers **b** proteasome subunit protein and mRNA levels. Quantification of the immunoreactive bands and of whole protein content was performed with the Image Lab software. Both total proteins and β-actin were used as loading control since β-actin levels were not significantly different between the cell lines. mRNA levels were determined by Real-time PCR and normalized on β2-microglobulin. The normalized signal for each subunit was expressed as fold change relative to 23132/87 cells. Bars are the mean ± SD of the values obtained in at least three independent extracts for protein and mRNA level quantification, respectively, using different batches of cells. *p < 0.05; **p < 0.01, ***p < 0.001 vs α subunits for proteins and vs 23132/87 ΔCt values for mRNAs

Protein expression levels of constitutive and immunoproteasome catalytic subunits were investigated by SDS PAGE and western immunoblotting analysis of whole cell extracts. MKN45 displayed higher β1i, β2i, β5i levels, and similar β1, β2, β5 content when compared to 23132/87 cells (Fig. 1a, b).

KATO III subunit expression essentially followed the same trend of MKN45, although lower levels of β5i and β2i compared to MKN45 were detected. β7 and α-type subunits which are present both in the CP and IP catalytic complexes were also analyzed and their levels found comparable in all the cell lines. The mRNA expression analysis indicated that both PSMB9 (β1i), PSMB10 (β2i) and PSMB8 (β5i) genes were up-regulated in MKN45 and KATO III compared to 23132/87 (Fig. 1b). Although differences in PSMB4 (β7) and PSMB5 (β5) mRNA could be detected, they did not correspond to changes in protein expression.

While expression of β5i and β1i together with constitutive proteasome subunits is necessary to form mixed proteasomes (Guillaume et al. 2010; Abi Habib et al. 2022), co-expression of the β2i subunit is essential to build immunoproteasomes. Thus, the concerted induction of all the three βi subunits suggests that both intermediate and immunoproteasome complexes can be potentially assembled in GC cells.

### Immunoproteasome subunits are incorporated into capped proteasome complexes

**Fig. 2:**
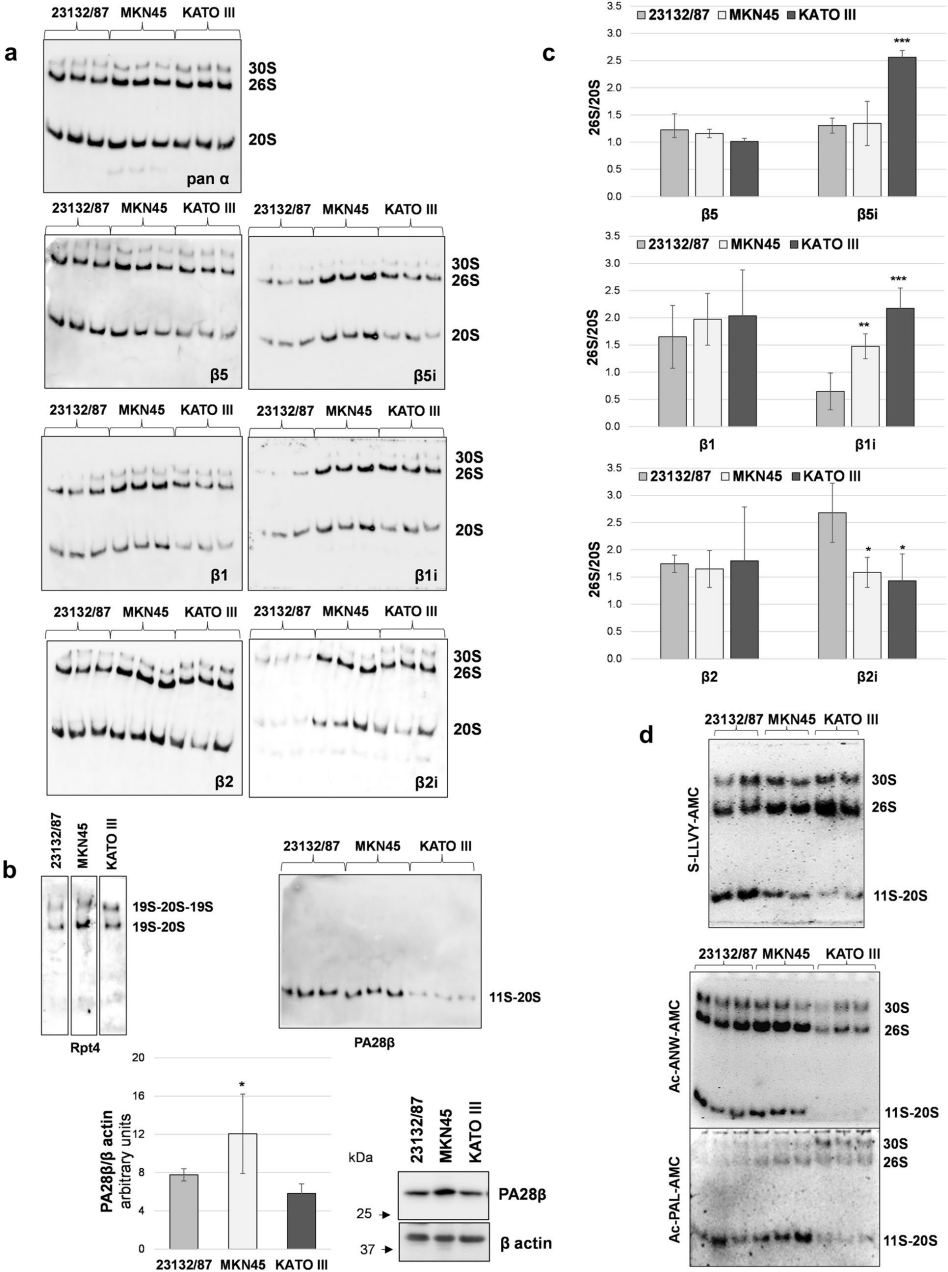
Subunit incorporation into proteasome complexes and in gel activity. **a** native PAGE separation of proteasome complexes; twenty µg of proteins from cell extracts obtained in the presence of ATP was loaded in each lane. After separation, proteins were denatured, transferred onto PVDF membranes and immunoblotted with the indicated antibodies. Three different extracts obtained from different cell batches are shown for each cell line **b** identity of the 30S and 26S complexes was confirmed by staining with a 19S-specific antibody, while an anti PA28β antibody was used to detected 11S-capped proteasomes. PA28β expression levels were assessed in cell extracts by SDS-PAGE and western immunoblotting analysis. Immunoreactive bands were visualized in a ChemiDoc system and quantified with the Image Lab software. PA28β/β actin ratio is shown in the graph (*n* = 3); *p < 0.05 versus 23132/87 cells **c** the abundance of β5, β5i, β1 and β1i, β2 and β2i subunits within 26S complexes relative to 20S complexes (26S/20S) was calculated after normalization of the specific signal on the corresponding pan α content in each sample, for each complex. Bars are the mean ± SD of the values obtained in 3 different extracts run in parallel. *p < 0.05, **p < 0.01, ***p < 0.001 vs 23132/87 **d** in gel peptidase activity using fluorogenic substrates for the chymotrypsin-like (s-LLVY-AMC), β5i (Ac-ANW-PAL) and β1i (Ac-PAL-AMC) activities. After complex separation by native PAGE, gels were incubated with the specific substrates indicated and activities detected in a Gel Doc system under UV light

To verify the effective incorporation of the immunosubunits into active proteasome complexes, native cell extracts were analyzed. Proteins were separated by native PAGE followed by in gel activity assay and immunoblotting with subunit-specific antibodies (Fig. 2a).

Three major complexes were detected after pan α subunit antibody staining as follows: the slower migrating one that corresponds to the 19S doubly capped 20S proteasome (30S); the medium band consisting of the singly capped 20S (26S); and the faster one shows the 20S proteasomes. The identity of the upper complexes was demonstrated by staining with an antibody against the AAA-ATPase subunit Rpt4 of the 19S regulatory complex (Fig. 2b). By contrast, immunostaining with an anti PA28β antibody revealed that at least part of the 20S complex was associated with the 11S regulator in 23132/87 and MKN45 cells and to a lesser extent in KATO III cells. Indeed, despite a similar 20S content, as revealed by the pan α antibody, the PA28β signal was markedly lower. Therefore, in our experimental conditions, the lower band corresponds to a combination of 11S capped and uncapped 20S proteasome complexes which comigrate. Besides, SDS-PAGE analysis of cell extracts revealed that only MKN45 had a significantly higher content of PA28β compared to 23132/87 cells, while no differences were found with KATO III cells (Fig. 2b). Thus, differential interaction of the 20S with the 11S complex seems not to be caused by higher or lower expression of the subunits forming the complex. By using specific antibodies, the incorporation of the immunosubunits within assembled proteasomes was demonstrated and shown to roughly reflect their relative cell expression levels (Fig. 2a). Specifically, β5i, β1i and β2i were particularly abundant in MKN45 and KATO III cell lines compared to 23132/87. Quantification of βi-type signal associated to each complex and normalization on its respective pan α signal, clearly demonstrated that the relative 26S over 20S βi content was significantly higher in MKN45 (β1i) and KATO III (β5i and β1i) compared to 23132/87 cells. Conversely no significant difference was found for their constitutive counterparts (Fig. 2c). Native gel analysis followed by in gel s-LLVY-AMC activity assay showed that most of the chymotrypsin-like activity was associated with 26S complexes in MKN45 and KATO III cells, while in 23132/87 was equally distributed between 11S-20S, 26S and 30S complexes (Fig. 2d). The appearance and/or prevalence of activity associated with 19S-capped complexes in MKN45 and KATO III could be also appreciated in assays where Ac-PAL-AMC (β1i-specific) and Ac-ANW-AMC (β5i-specific) substrates were used for in gel activity (Fig. 2d). Since uncapped proteasome complexes are usually inactive (Shibatani and Ward 1995), the low 20S-associated activity found in KATO III cells is in agreement with our previous observation that in this cell line most of the 20S particles exists as free entities. The finding that in KATO III cells most of the immunoproteasome subunits are incorporated into 20S complexes associated with the 19S rather than with the 11S regulator (Fig. 2b, c) is in contrast with the evidence of Fabre et al (2015) that 20S immunoproteasomes and hybrid proteasomes preferentially associate with the 11S complex. On the other hand, more recent literature has provided evidence that constitutive and immunoproteasome 20S particles have the same affinity for the 11S particle (Schmidtke et al. 2019). The authors suggest that these interactions may be modulated by post translational modifications which may occur in a cell-type and context specific manner. In support to this hypothesis the binding of PA28αβ to the 20S proteasome has been shown to increase in oxidative stress conditions (Abi Habib et al. 2020), highlighting that 20S interactions with the 11S particle may be more complex/dynamic than previously assumed.

**Fig. 3:**
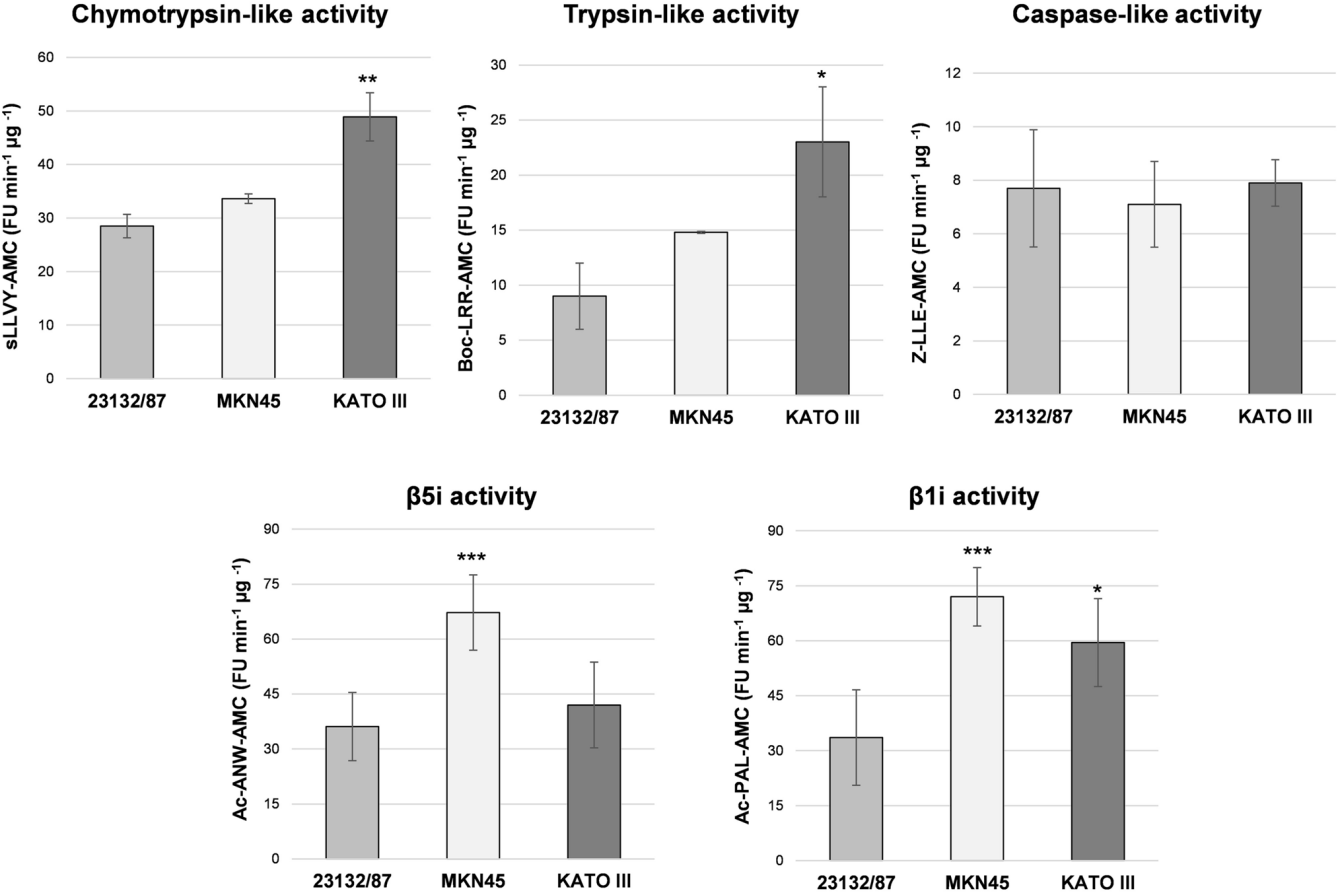
Activity assays with fluorogenic substrates. Proteasome activities were assayed in cell extracts using fluorogenic substrates for the chymotrypsin-like (s-LLVY-AMC), trypsin-like (Boc-LRR-AMC), caspase-like (Z-LLE-AMC) as well as for the β5i (Ac-ANW-AMC) and β1i-associated (Ac-PAL-AMC) activities. Fluorescence intensity was measured in a fluorimeter and activity was expressed as arbitrary fluorescence units (FU) min^−1^ µg^−1^ of total proteins. Bars represent the mean ± SD of the values obtained in at least three extracts deriving from different batches of cells. *p < 0.05; **p < 0.01; ***p < 0.001 vs 23132/87

The overall proteasome activity associated with the distinct proteasome patterns observed was investigated using fluorogenic peptide substrates in cell-free extracts. KATO III displayed a profile with a significantly higher chymotrypsin-like and trypsin-like activity compared to the extracts obtained from the other cell lines, suggesting that they may be particularly abundant in mixed proteasomes (Fig. 3). In addition, higher β1i and β1i/β5i activities were found in KATO III and MKN45, respectively, compared to 23132/87 cells.

### GC proteasome patterns are associated with sensitivity to proteasome/immunoproteasome inhibitors

**Fig. 4:**
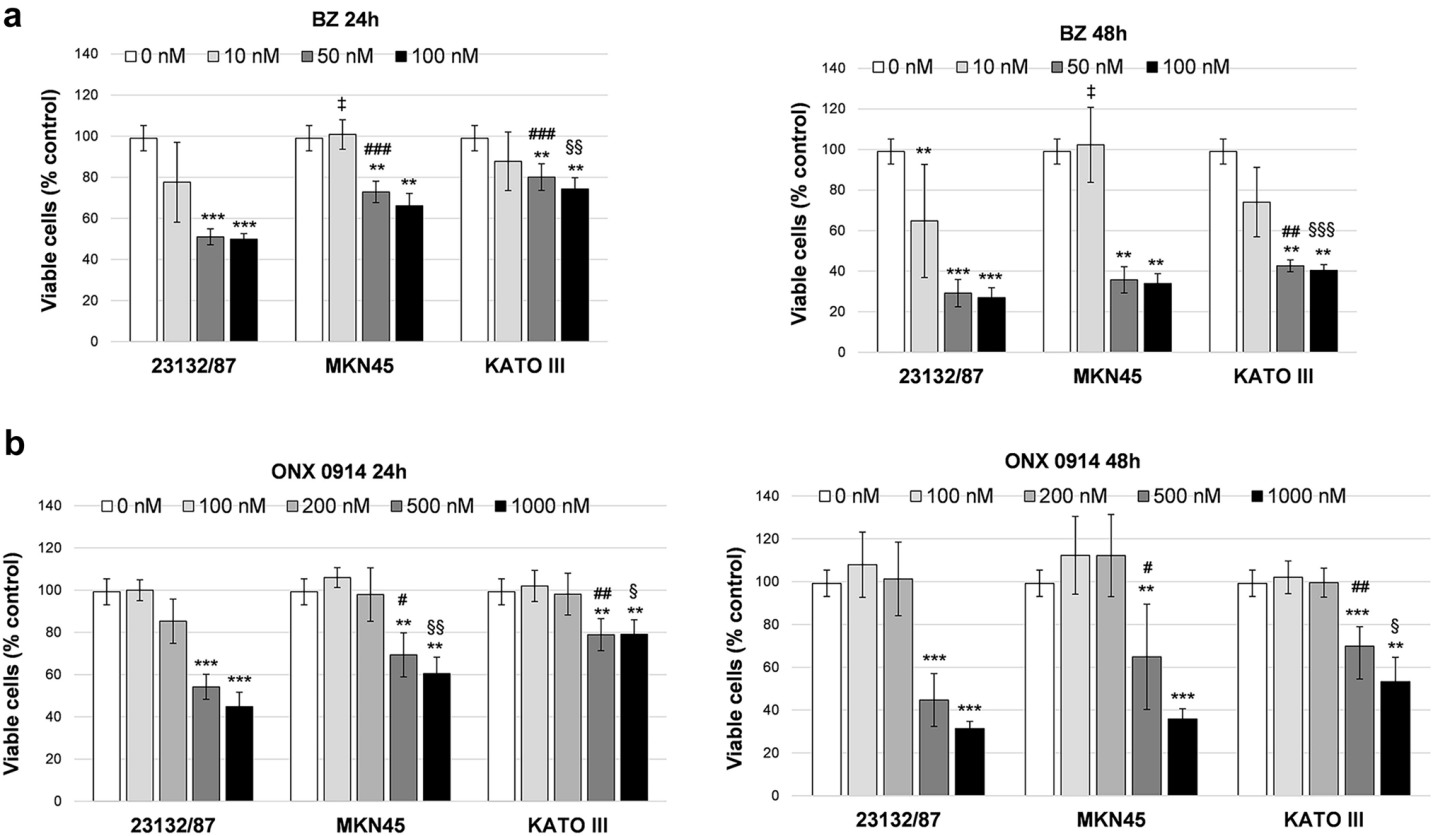
Viability assay in cells treated with proteasome inhibitors Bortezomib and ONX 0914**.** Cells were incubated with different concentrations of Bortezomib (BZ) **(a)** and ONX 0914 **(b)** for 24 h and 48 h. Cell viability was assessed by a tetrazolium salt-based proliferation assay. Viable cells in treated samples were calculated as percent of the control value obtained in cells incubated with the highest concentration of DMSO used (0 nM). Bars represent the mean ± SD of the values obtained in at least three independent experiments. **p < 0.01, ***p < 0.001 vs the relative control value; ‡*p* < 0.05; #*p* < 0.05, ##*p* < 0.01, ###*p* < 0.001; § < 0.05 §§ p < 0.01, §§§* p* < 0.001, vs 23132/87 cells at the same inhibitor concentration

To investigate the role of the different proteasome asset in cancer cell viability, GC cells were treated with the FDA-approved proteasome inhibitor Bortezomib (BZ) and the immunoproteasome inhibitor ONX 0914. Different concentrations of the inhibitors were tested to determine the cytotoxic dose. BZ displayed a strong cytotoxic activity towards 23132/87 cells at 50 nM and 100 nM concentration with a 50% and 25% of viable cells after 24 h and 48 h incubation, respectively. At these concentrations, a significantly higher number of viable cells was present in MKN45 and KATO III at 24 h, and in KATO III at 48 h compared to 23132/87 (Fig. 4a), demonstrating that KATO III are the most resistant to BZ, followed by MKN45 and 23132/87. Cytotoxicity with the immunoproteasome inhibitor ONX 0914 was observed at 500 nM and essentially followed the same trend of BZ with KATO III being the less affected followed by MKN45 and 23132/87 (Fig. 4b).

**Fig. 5:**
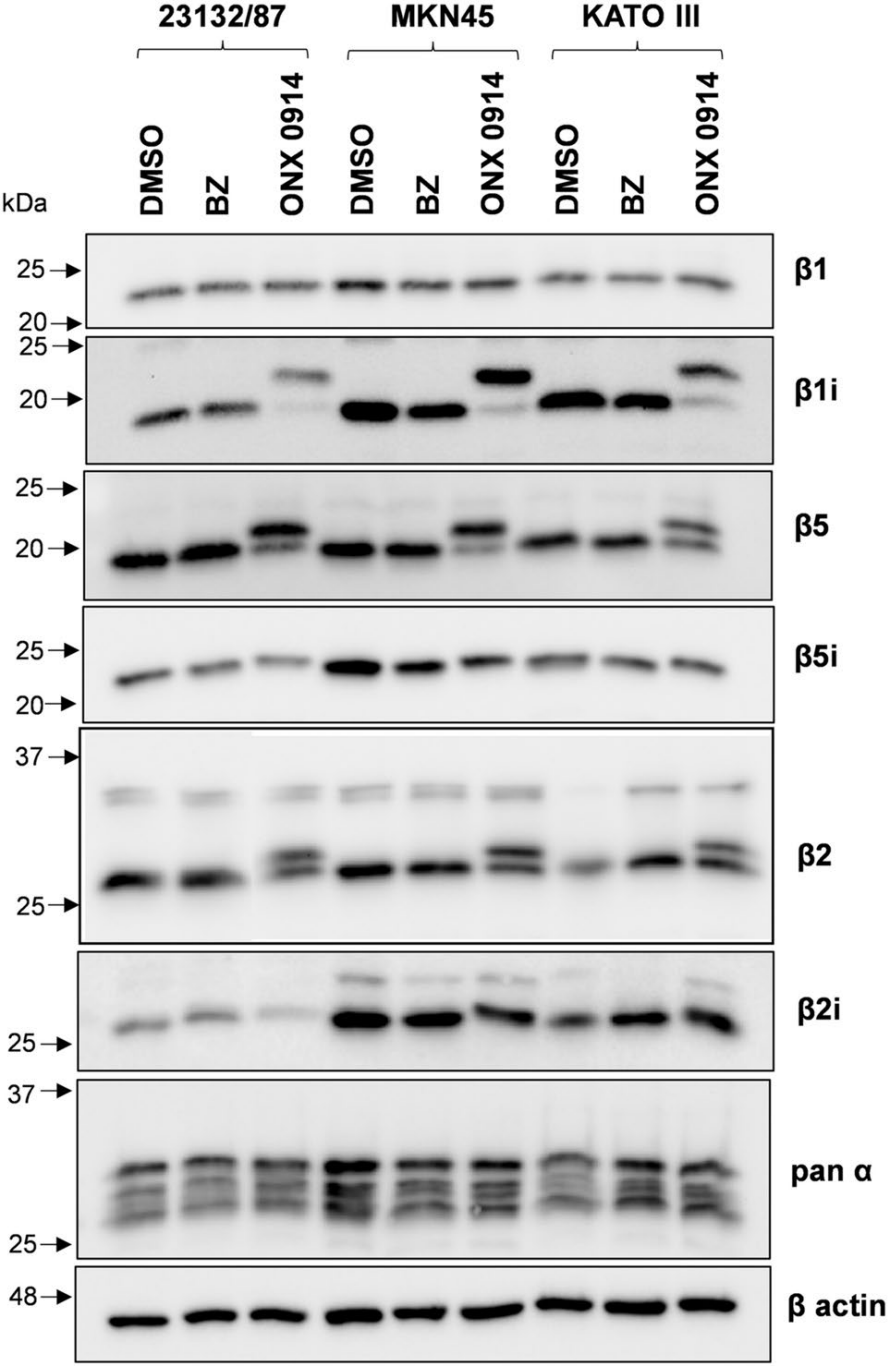
Proteasome subunit expression after proteasome inhibition with Bortezomib and ONX 0914. Cell extracts (2.5 μg protein) from cells treated with 50 nM Bortezomib (BZ) or 500 nM ONX 0914 for 16 h were separated onto 12% (w/v) SDS polyacrylamide gels, electroblotted and immunoblotted with the antibodies indicated in the figure. As control, cells received only the vehicle DMSO. β-actin was stained as loading control

To verify effective proteasome/immunoproteasome inhibition, cell extracts from treated cells were assayed with fluorogenic substrates. For these experiments we selected inhibitor concentrations corresponding to the lowest dose able to produce a significant drop in cell viability (i.e. 50 nM for BZ and 500 nM for ONX 0914). Both BZ and ONX 0914 strongly reduced chymotrypsin-like and β5i and β1i-associated activities. The trypsin-like and caspase like activities were affected as well, although to a lesser extent, except for BZ which more markedly reduced the caspase-like activity (Fig. S1). High concentrations of BZ have been demonstrated to inhibit β1 and β2 activities as well as immunoproteasome subunit activities (Demo et al. 2007; Crawford et al. 2006). On the other hand, multiple evidence indicates that at least two active sites need to be inhibited to achieve cell apoptosis which would explain the efficacy of inhibitors co-inhibiting more than one site (Britton et al. 2009; Weyburne et al. 2017). Interestingly, subunit expression did not change at the protein level after BZ treatment. By contrast, β5, β2 and β1i subunits levels decreased in cells incubated with ONX 0914 and a higher molecular weight specie appeared for each subunit (Fig. 5).

Proteasome subunits are synthesized as precursors which are subsequently processed into mature forms (Heinemeyer et al. 1997). However, the slower migrating bands are consistent with the molecular weight of the precursor only in the case of β1i (23 kDa), but not β2 (30 kDa) and β5 (28 kDa). In addition, proteasome subunits are targets of post-translational modifications, including irreversible binding of ONX 0914 to catalytically active subunits resulting in altered electrophoretic mobility in SDS-PAGE (Basler et al. 2018). To discriminate between partially processed and covalently modified β subunits, cells were incubated with different concentrations of ONX 0914 in the presence of a translational inhibitor. Western immunoblotting analysis clearly indicates that accumulation of higher molecular weight species was dose-dependent and cycloheximide treatment did not inhibit their formation (Fig. S2).

### ONX 0914 affects cell migration in metastatic GC cells

**Fig. 6:**
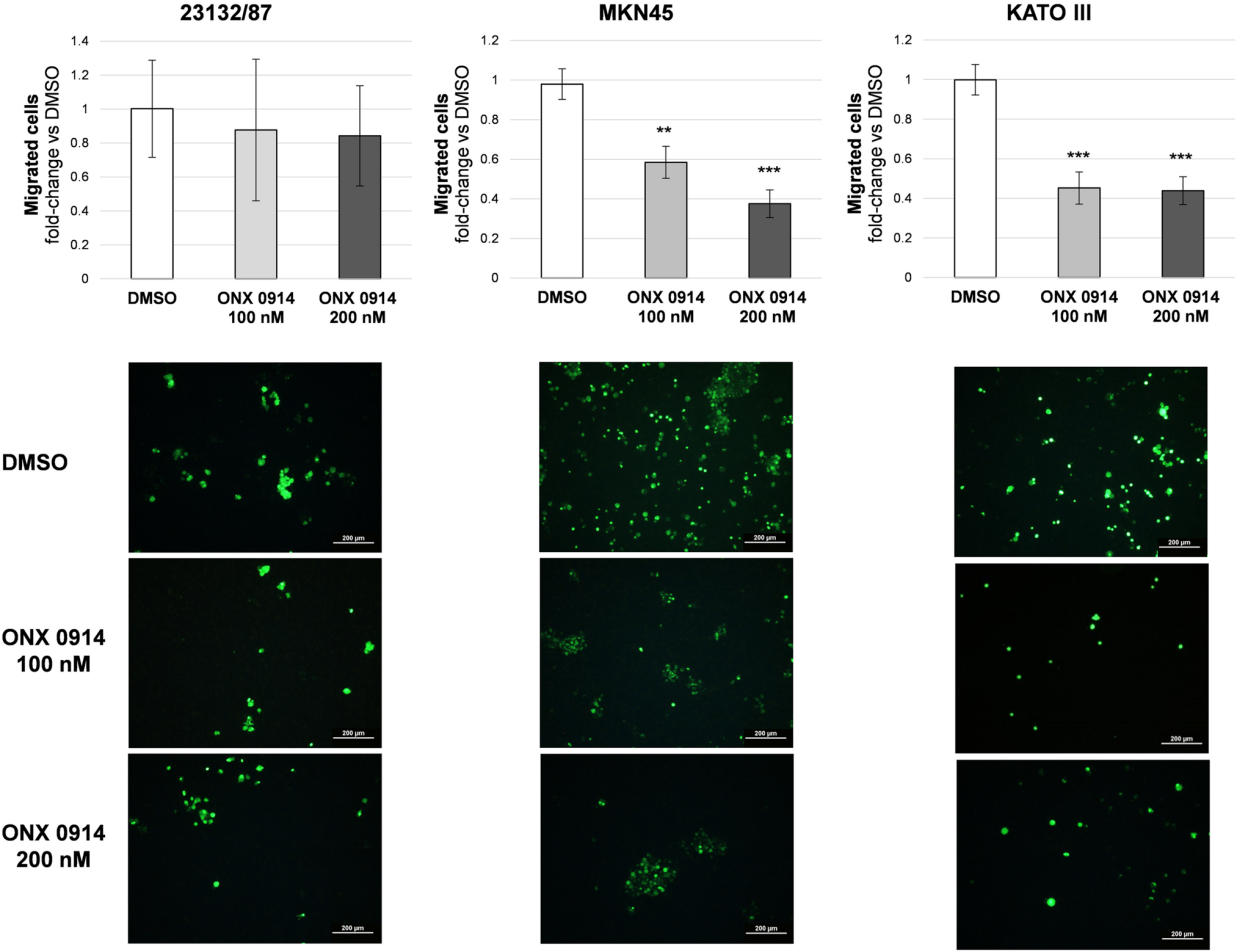
Transwell migration assay. Cells were seeded on the upper side of the transwell membrane. In the lower compartment 10% (v/v) FBS was added as chemoattractant. ONX 0914 was added to the lower compartment at non-toxic concentrations (i.e. 100 and 200 nM). After 24 h incubation, cells were counted from 10 random microscope fields for each sample in 3 independent experiments and expressed as fold change vs control condition (DMSO). Values are means ± SD. **p < 0.01, ***p < 0.001 vs DMSO. Representative images of calcein-labelled cells captured after 24 h of treatment are shown in the bottom panel. Scale bar: 200 µm

To study whether incorporation of immunosubunits into proteasomal complexes may have a role in GC cell migration, cells were treated with non-cytotoxic doses of ONX 0914, i.e. 100 and 200 nM for 24 h. Western immunoblotting analysis and activity assays in cell extracts from treated cells clearly demonstrated that 100 nM ONX 0914 was still sufficient to inhibit most of the β5i-associated activity, and 60% of the β1i activity, while it displayed more limited or modest effects on the other activities (Fig. S3), suggesting that at this dose the inhibitor is more selective for the immunoproteasome compared to the cytotoxic dose of 500 nM (Fig. S1). In agreement with this observation only 500 nM ONX 0914 led to massive accumulation of ubiquitin-conjugated proteins (Fig. S4). The transwell assay was performed to investigate the anti-chemotactic ability of ONX 0914 on GC cell lines. The inhibitor did not affect 23132/87 migration, while significantly reduced MKN45 and KATO III cell migration through the cell culture insert (Fig. 6).

## Discussion

Gastric cancer is among the most common malignancy worldwide (Sitarz et al. 2018; Smyth et al. 2020). Chemotherapeutics are employed to treat unresectable or metastatic gastric adenocarcinoma representing 90–95% of gastric carcinoma, however the overall prognosis remains poor. Therefore, there is a certain need to identify new molecular pathways involved in GC pathogenesis to develop prognostic markers and find new drug targets.

Multiple components of the Ubiquitin-dependent system with oncogenic or tumor-suppressing activity have been described as involved in GC (Zhong and Huang 2016). Proteasome inhibitors have also been tested as therapeutics in GC and overexpression of different proteasome subunits has been correlated with poor prognosis (Nakata et al. 2011; Kwon et al. 2016). However, structure and function of the proteasome in GC remain poorly investigated. Previous studies were based on the knockdown of single proteasome components, an approach that, although informative, doesn’t consider the complexity and plasticity of this proteolytic machinery. Indeed, the levels of functional proteasomes depends not only on subunit expression but also on their quantitative and qualitative assembly into proteasome complexes whose activity can be further regulated by capping of the 20S core with different regulatory particles. Moreover, proteasomes are reversibly disassembled and reassembled, configurations changed and subunits post-translationally modified to meet changing proteolytic needs or in response to inhibitors (Glickman and Raveh 2005; Welk et al. 2016).

Here we show that GC cell lines express different levels of immunoproteasome subunits assembled into differently capped proteolytic particles giving rise to proteasomal repertoires which can be associated with sensitivity to proteasome inhibitors and migratory capacity of GC cells.

Early and advanced gastric cancer is characterized by marked morphological and molecular changes leading to diffuse and intestinal-type gastric carcinoma. The two types share common pathways but display also marked differences (Tanabe et al. 2020). The molecular basis of these divergences must be still elucidated. Our results demonstrate that cells with diffuse-type traits express high levels of immunoproteasome subunits which are preferentially incorporated into 19S-capped complexes. These complexes represent the predominant catalytically active proteasome species in these cells. By contrast, in cells with epithelial-type phenotype immunoproteasome subunits are less expressed and equally distributed between PA28- and 19S-capped 20S proteasome complexes all of which contribute to the overall proteasome activity. In solid and hematological tumors low immunoproteasome subunit expression has been related to resistance to proteasome inhibition (Tripathi et al. 2021). However, this is not the case of GC where resistant cells appear to be those more dependent on 19S-capped complex activity rather than those expressing the highest immunoproteasome subunit levels. Interestingly, assembly of the 26S complex was found increased in intestinal tumors where it promotes tumorigenesis (Levin et al. 2018). Moreover, it has been demonstrated that after Ras transformation, immortalized cell lines elevate the levels of the 26S proteasome, an event which is crucial for their survival (Tsvetkov et al. 2018). Overall, these observations indicate that oncogenic transformation is highly dependent on 26S proteasome function, making the 19S regulatory particle an attractive target for therapeutic interventions. There is little information on whether specific proteasome forms with different regulatory caps are more critical than others. If the importance of this regulator will be confirmed in GC, targeting of the 19S particle may provide a more efficient approach for treating diffuse-type gastric cancers compared to proteasome/immunoproteasome inhibition.

Another interesting observation relates to the evidence that cells deriving from primary tumors (i.e. 23132/87) express lower levels of proteasome immunosubunits compared to metastatic ones (MKN45 and KATO III). When exposed to non-cytotoxic doses of the immunoproteasome inhibitor ONX 0914, a marked reduction of cell migration was observed in metastatic cell lines, suggesting that transcriptional up-regulation of the immunosubunits is linked to the metastatization process. This result agrees with the observation that PSMB8 knockdown reduces GC cell line migration and invasion, but not proliferation; the molecular mechanisms are presently unknown (Kwon et al. 2016). Similar results were obtained in breast cancer cells where silencing of β5i or PA28α/β coding genes resulted in a marked inhibition of their invasiveness and migration ability without affecting the proliferation rate. The authors identified cyclin-dependent kinase 15 (CDK15) as negative regulator of cancer cell motility and as a possible target of the 11S regulator and immunoproteasome (Li et al. 2019). PSMB8 regulation of cell growth and migration, possibly via ERK1/2 (extracellular signal-regulated protein kinase) and AKT (serine/threonine kinase 1) signaling, was also observed in glioma cells (Yang et al. 2018). Therefore, evidence is accumulating pointing to a role of the immunoproteasome in promoting invasion and metastasis. In this context, specific pharmacological targeting of the immunoproteasome is thus expected to be effective for the treatment of metastatic cancers. Here we provide evidence that in GC ONX 0914 can induce the post-translational modification of β1i, probably accounting for β1i inhibitory activity, but also of β2 and β5 constitutive subunits, which may result in a wider impact of this inhibitor on proteasome function. Activity assays using fluorogenic substrates further support this evidence. The inability to see the covalent attachment of ONX 0914 to β5i, which is the main target of the inhibitor, could be due to insufficient PAGE resolution being the resulting mobility shift in this case very modest. A lower selectivity of ONX 0914 in non-immune cells has been reported also by others (Neumaier et al. 2020). Inhibition of constitutive proteasome is thought to affect proteostasis and induce toxicity; accordingly, only high dose of ONX 0914 resulted in CP inhibition and accumulation of ubiquitin–protein conjugates, in agreement with the cytotoxic effects exerted at this dosage. By contrast, low dose inhibited migration without affecting ubiquitin pools which supports a more specific targeting of the immunoproteasome.

In conclusion, data presented in this paper suggest that aggressiveness of diffuse-type GC may be related to high expression of immunoproteasome subunits together with their preferential incorporation into catalytically active19S-capped proteolytic complexes. This observation has important implications in developing therapeutic strategies targeting the proteasome.

## Supplementary Information

Below is the link to the electronic supplementary material.

Supplementary file1 (PDF 534 KB)

## Data Availability

Most data generated or analyzed during this study are included in this article. The materials used and/or analyzed during the current study are available from the corresponding author on reasonable request.

## Abbreviations

AMC: 7-Amido-4-methylcoumarin
BZ: Bortezomib
CHX: Cycloheximide
CP: Constitutive proteasome
DMSO: Dimethyl sulfoxide
DTT: Dithiothreitol
GC: Gastric cancer
i20S: Immunoproteasome 20S
IP: Immunoproteasome
PVDF: Polyvinylidene difluoride
STR: Short tandem repeat
Ub: Ubiquitin
