# Supplementary material for: Gastric cancer cell types display distinct proteasome/immunoproteasome patterns associated with migration and resistance to proteasome inhibitors
Source: J Cancer Res Clin Oncol. 2023 Jun 1;149(12):10085–97. doi: 10.1007/s00432-023-04948-z (PMC10423134; doi:10.1007/s00432-023-04948-z)
Supplement: Supplementary file 1 — Supplementary file1 (PDF 534 KB) [file 432_2023_4948_MOESM1_ESM.pdf]

Journal of cancer research and clinical oncology

Gastric cancer cell types display distinct proteasome/immunoproteasome patterns associated with migration and resistance to proteasome inhibitors

Francesca Monittola§, Marzia Bianchi§, Maria Gemma Nasoni, Francesca Luchetti, Mauro Magnani, Rita Crinelli*

Department of Biomolecular Sciences, University of Urbino Carlo Bo, 61029 Urbino (PU), Italy

§equally contributed

*Corresponding author—Rita Crinelli

e-mail: rita.crinelli@uniurb.it

ORCID: 0000-0002-1235-8910

SUPPLEMENTARY FIGURES

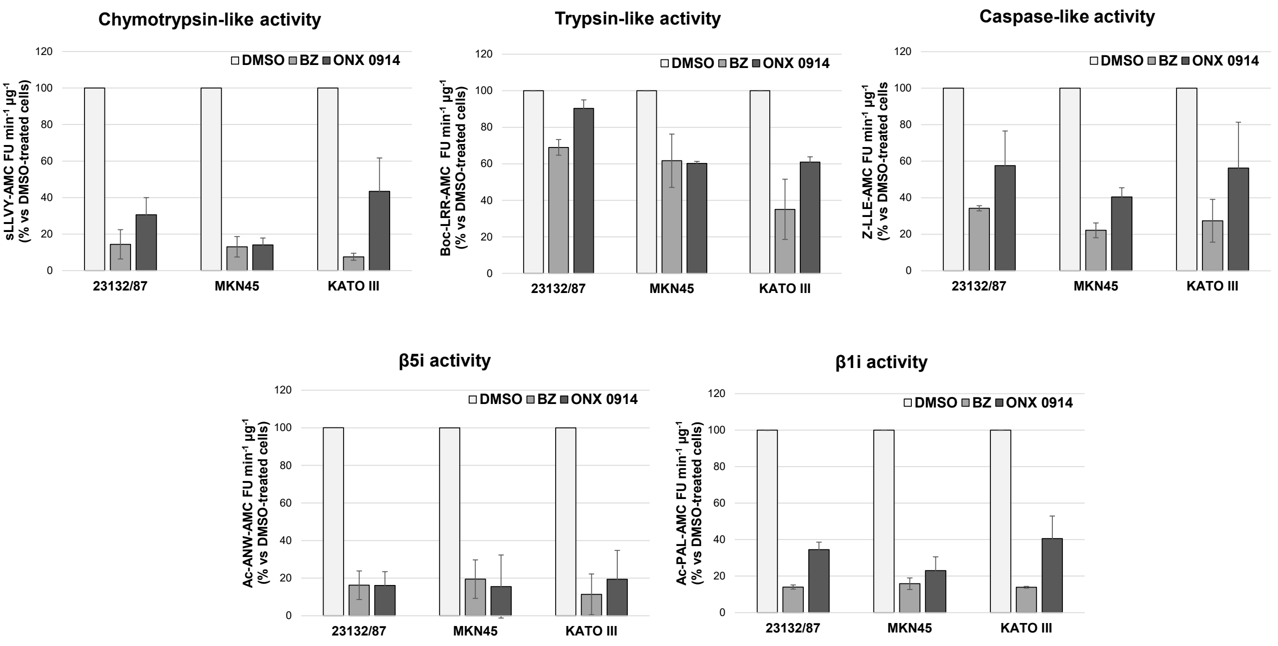

Fig. S1 Proteasome and immunoproteasome activity in cell extracts obtained from cells treated with a toxic dose of BZ (50 nM) or ONX 0914 (500 nM) for 24h. Values are expressed as % of the control value (DMSO). Bars represent the mean ± SD of the values obtained in n=2 independent experiments

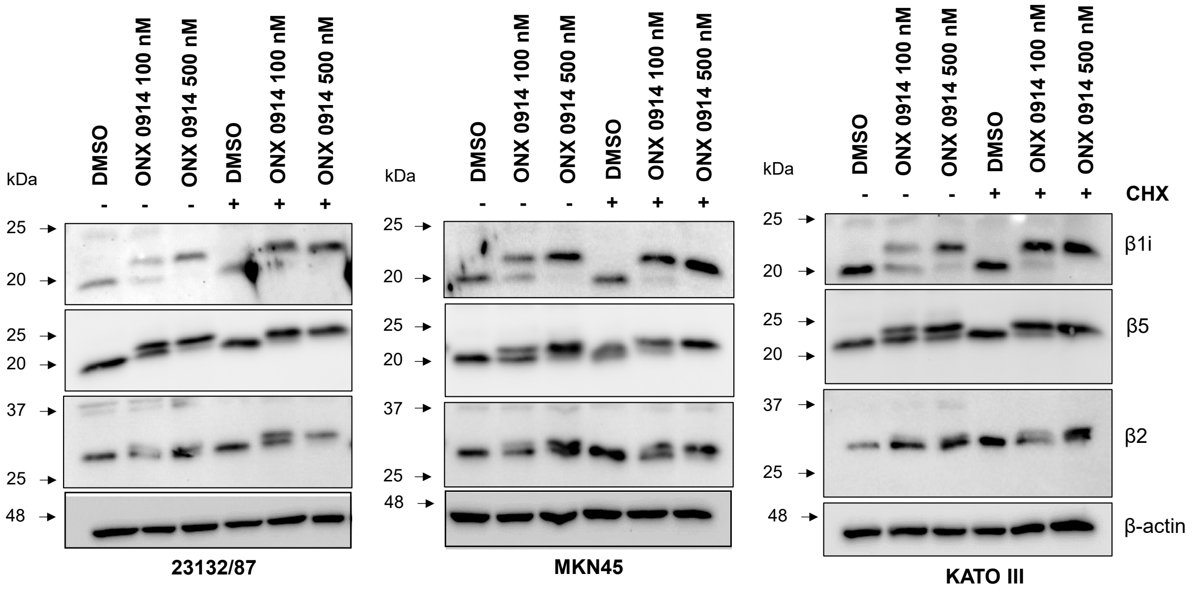

Fig. S2 Western immunoblotting analysis of cell extracts treated with ONX 0914 in the absence and presence of 50 µg/ml CHX for 16h

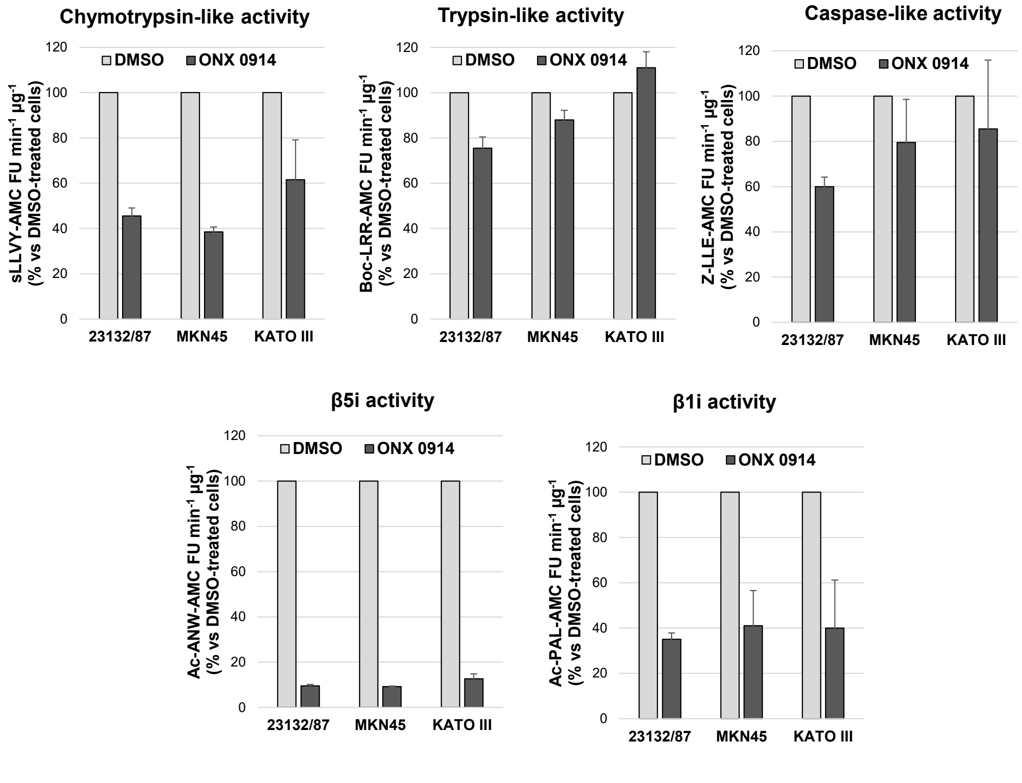

Fig. S3 Proteasome and immunoproteasome activity in cell-free extracts obtained from cells treated for 24h with a non-toxic dose of ONX 0914 (100 nM). Values are expressed as % of the control value (DMSO). Bars represent the mean ± SD of the values obtained in n=2 independent experiments

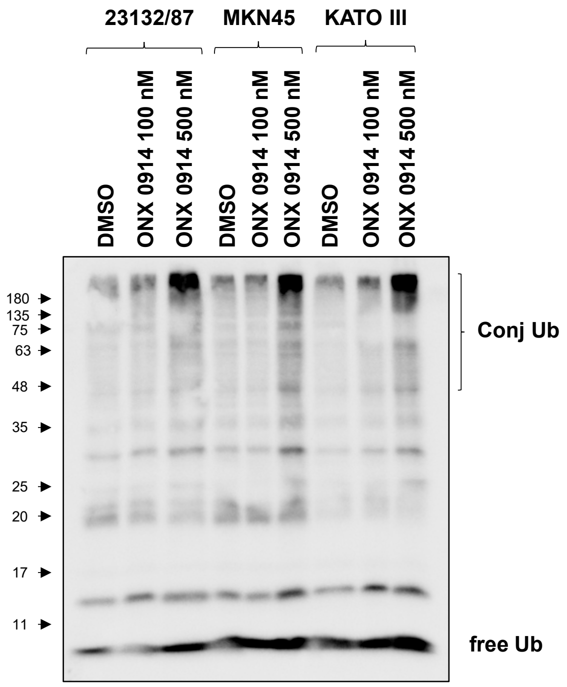

Fig. S4 Ubiquitin and Ub-conjugated proteins in ONX 0914-treated GC cells. Whole cell extracts (5 µg) were resolved on a 13% (w/v) polyacrylamide gel and immunoblotted with an antibody against ubiquitin (Ub). On the left, arrows indicate the position of molecular weight markers
